# DYADIC COPING AND WELL-BEING AMONG COUPLES TAKING CARE OF EACH OTHER

**DOI:** 10.1093/geroni/igac059.2350

**Published:** 2022-12-20

**Authors:** Vivian Wei Qun Lou, Huiying Liu, Wenmiao Wu

**Affiliations:** The University of Hong Kong, Hong Kong, Hong Kong; Central South University, Chang Sha, Hunan, China (People's Republic); The University of Hong Kong, Hong Kong, Hong Kong

## Abstract

**Background:**

In the context of population aging, more and more couples would have the chance to taking care of each other when one or both of them experience functioning decline. Objective: Taking a dyadic perspective, the objective of this study is to examine collaborative dynamics in mastering daily stressors, and the relationship between couples’ practice of dyadic coping and individual well-being. Method: A total of 77 dyads were recruited from community elderly centres located in four different geographic locations of Hong Kong. Potential participants who met the following inclusion criteria: (1) both partners aged 60 or above; (2) both partners living alone in the same household; (3) one or both partners reporting at least one limitation in performing ADLs and/or IADLs, were invited to complete a survey questionnaire interviewed by trained researchers. Standardized measures by Dyadic Coping Inventory (DCI), Centre for Epidemiological Studies-Depression Scale (CES-D), EQ-5D, and Relationship Assessment Scale (RAS), respectively. The Actor-Partner Interdependence Model (APIM) framework was used for data analysis. Findings: The most adopted collaborative coping strategies were non-negative dyadic coping of oneself, non-negative dyadic coping of the partner, and common dyadic coping. Supportive and delegated dyadic coping strategies were strongly associated with marital satisfaction and health-related quality of life.

**Conclusion:**

Collaborative dynamics among couples when one or both experience functional impairment is associated with quality of relationship. The findings advocate a shift from individual-based stress process on caregiving to a dyadic-based process that address joint needs of both spouses in super-aging societies globally.Funding: RGC 17605119

